# Supplementary material for: A Notch positive feedback in the intestinal stem cell niche is essential for stem cell self‐renewal
Source: Mol Syst Biol. 2017 Apr 1;13(4):927. doi: 10.15252/msb.20167324 (PMC5408779; doi:10.15252/msb.20167324)
Supplement: Supplementary file 1 — Expanded View Figures PDF [file MSB-13-927-s001.pdf]

Expanded View Figures

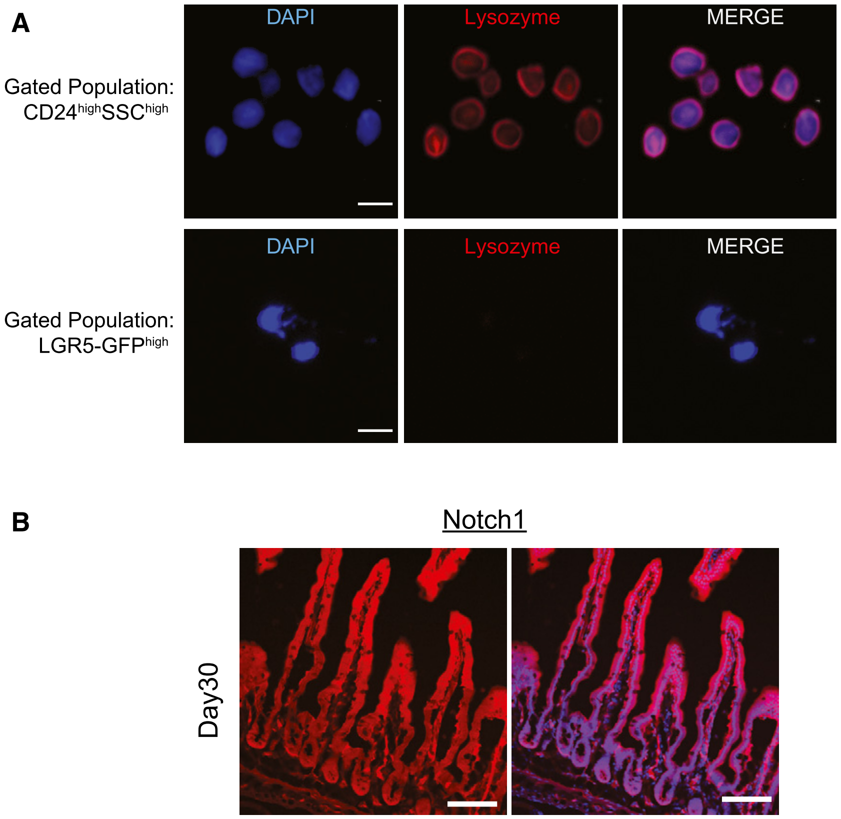

Figure EV1. Lysozyme expression in isolated Paneth cells and linage tracing of Notch1^{+} cells.

A Immunofluorescence (IF) image of sorted CD24^{high}/SSC^{high} cells and LGR5-GFP^{high} cells. CD24^{high}/SSC^{high} cells show expression of Paneth cell-specific marker lysozyme (red) exclusively. DAPI labels nuclei, and scale bar represents 10 μm.

B Representative images of intestinal tissue derived from tamoxifen-induced Notch1-CreERT2 KI × Rosa26-tdTomato-WPRE mice. Shown are images from 30 days post-tamoxifen induction. Notch1 IF (red). DAPI labels nuclei and scale bar represents 100 μm.

Figure EV2. Notch1 positive feedback in mouse intestine.

A ChIP-Seq signal of LICR histone tracks (H3K4me1 and H3K27ac) on mouse small intestine cells from ENCODE at UCSC Genome Browser. Left: H3K4me1 (top) and H3K27ac (bottom) occupancy related to Notch1. Right: H3K4me1 (top) and H3K27ac (bottom) occupancy related to Notch2.

B Top: Mouse Notch1 gene. Red line indicates the location of NICD/RBPJk binding motif on mouse Notch1. Bottom: Sequence and chromatogram of NICD binding motif in mouse Notch1 following ChIP-PCR from LGR5-EGFP^{+} CBCs.

C Agarose gel analysis of ChIP-PCR products from LGR5-EGFP^{+} CBCs validating NICD binding to the motif in Notch1 sequence. LGR5-EGFP^{+} CBCs were sorted from organoids treated with DMSO, DAPT, or JAG1.

D Organoids extracted from LGR5-EGFP × CreERT2/Rosa26-YFP-NICD mice were treated with tamoxifen to induce NICD overexpression (NICD-OE). Shown is agarose gel analysis of ChIP-PCR products to validate active NICD binding on Notch1.

E Representative sequences from selected organoid clones transfected with CRISPR/Cas9 gRNAs showing indel mutations in the targeted region of the mouse NICD binding motif. Yellow box represents the putative binding sequence region, where red indicates indel mutations by CRISPR.

F LGR5-EGFP^{+} CBCs were transfected with either an empty vector control or CRISPR/Cas9 gRNA and subsequently treated with DMSO, DAPT, or JAG1. Shown is ChIP-qPCR analysis of Notch1, indicating enrichment with NICD antibody compared with IgG control. The experiment was performed in triplicate and presented mean ± SEM (**$P \leq 0.01$; Student's *t*-test).

G RT–PCR measurements indicating Notch1 expression in LGR5-EGFP^{+} CBCs. Isolated single LGR5-EGFP^{+} CBCs were transfected with either an empty vector control or CRISPR/Cas9 gRNAs and subsequently treated with DMSO, DAPT, or JAG1 (embedded in Matrigel). The experiment was performed in triplicate and presented mean ± SEM (*$P \leq 0.05$, **$P \leq 0.01$; Student's *t*-test).

H Single LGR5-EGFP^{+} CBCs were transfected with either an empty vector control or CRISPR/Cas9 gRNAs and propagated as organoids. Shown is Western blot analysis for NICD expression in sorted LGR5-EGFP^{+} CBCs from each condition. Actin was used as a loading control.

I RT–PCR measurements indicating Notch1/2, Hes1/5, and Lgr5 expression in LGR5-EGFP^{+} ISCs for each condition described in (G). The experiment was performed in triplicate and presented mean ± SEM (**$P \leq 0.01$; Student's *t*-test).

J Single LGR5-EGFP^{+} CBCs were transfected with either an empty vector control or CRISPR/Cas9 gRNAs and propagated as organoids for 15 days. Shown are representative FACS plots for each condition including gated analysis to isolate CD24^{high}/SSC^{high} Paneth cells and LGR5-EGFP^{+} ISCs.

Source data are available online for this figure.

▶

© 2017 The Authors

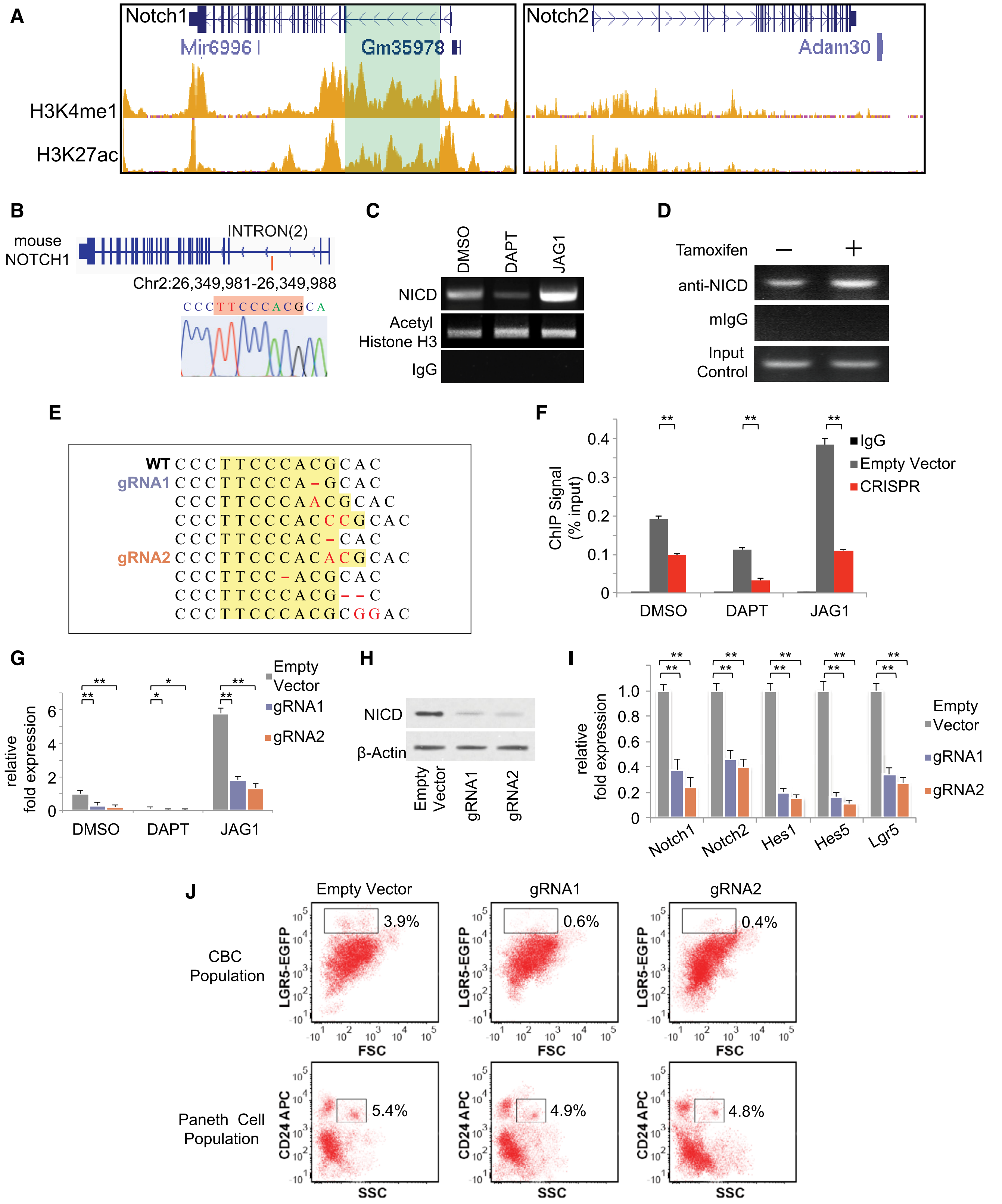

Figure EV2.

 © 2017 The Authors

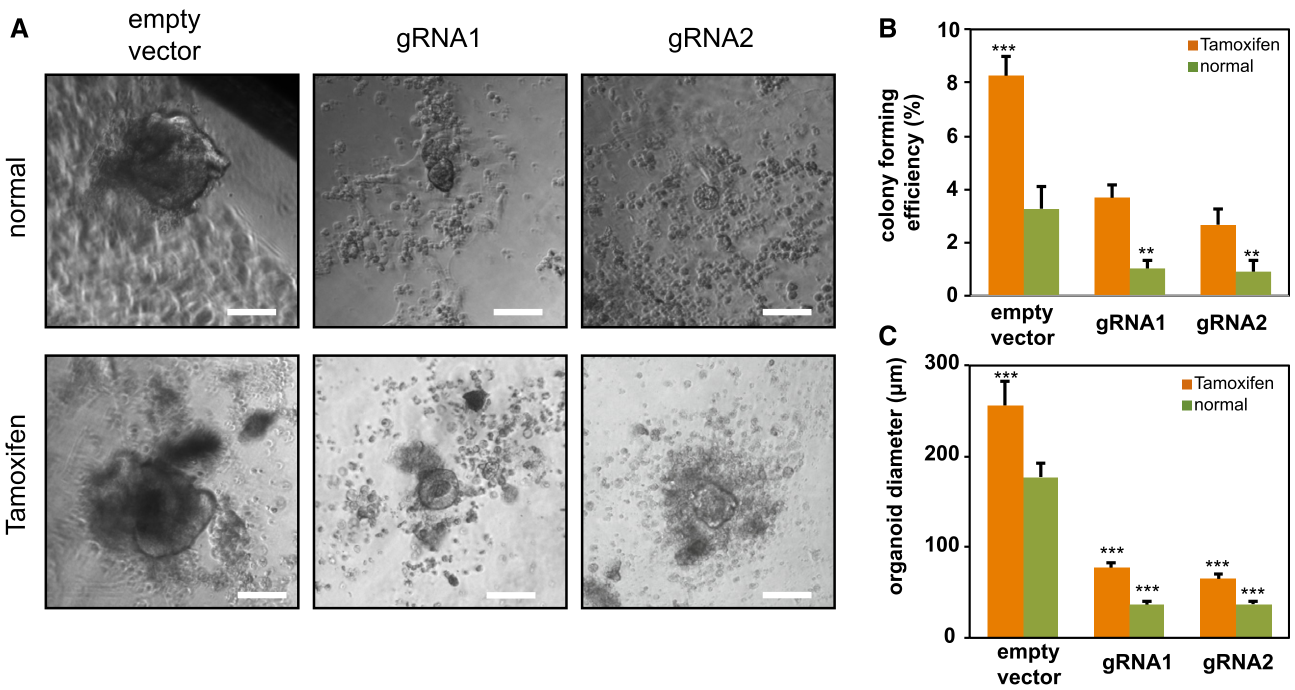

Figure EV3. Notch activation in Notch1 positive feedback knock-out intestine organoids.

Intestine cells extracted from LGR5-EGFP × CreERT2/Rosa26-YFP-NICD mice were treated with tamoxifen to induce NICD overexpression (NICD-OE) and subsequently transfected with either an empty vector (control) or CRISPR/Cas9 gRNAs.

A Shown are representative brightfield images over 3 weeks. Scale bar represents 100 μm.

B Colony-forming efficiency (n = 3) measured after 3 weeks. Quantitative analysis calculated from 1,000 cells/replicate.

C Quantitative comparison of organoid diameters (n = 5) after 3 weeks.

Data information: The experiment was performed in replicates and presented mean + SD (**$P \leq 0.01$, ***$P \leq 0.001$; Student's t-test compares other conditions to empty vector in normal condition separately).

© 2017 The Authors

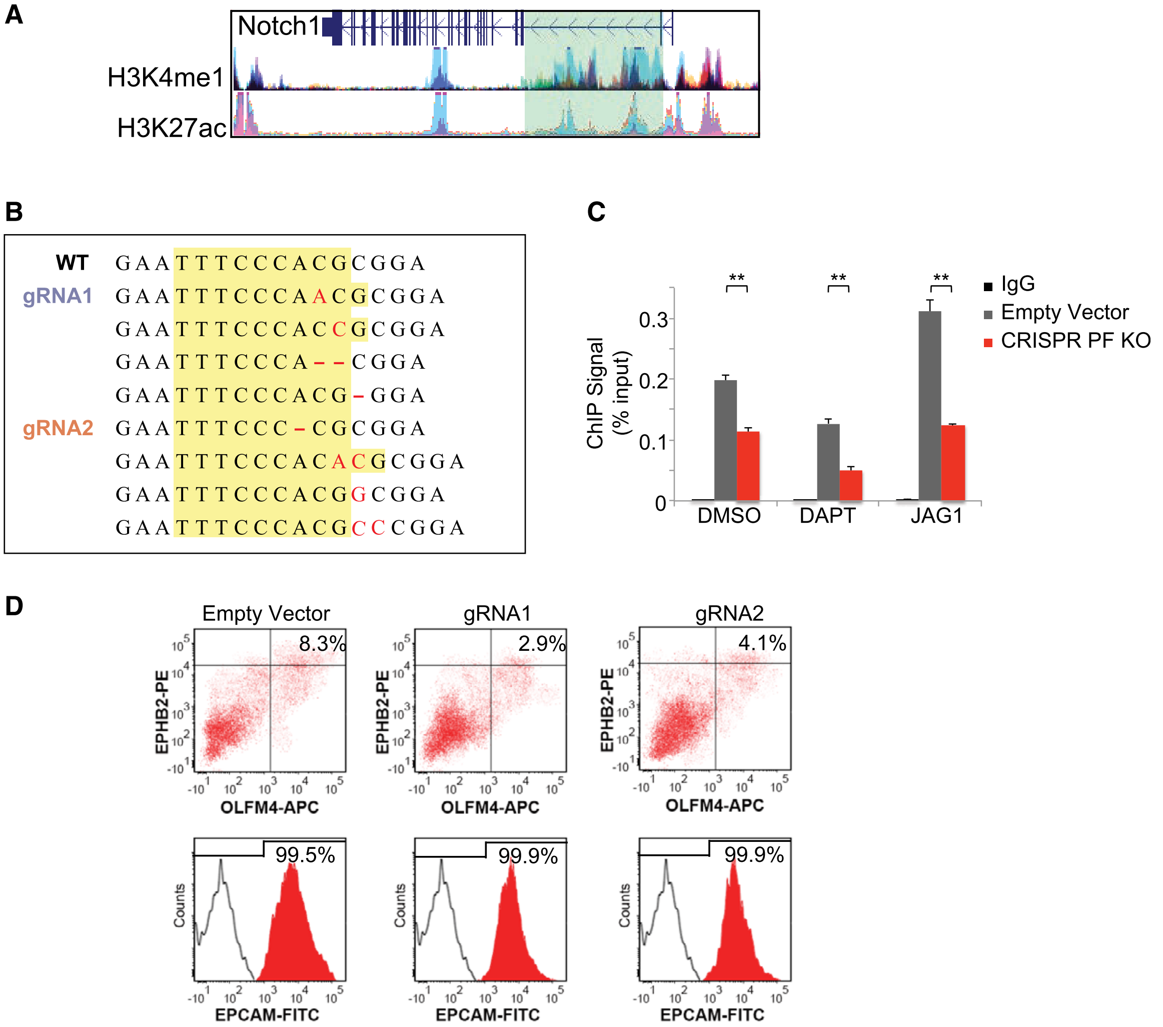

Figure EV4. Notch1 positive feedback in human colon stem cells.

A ChIP-Seq signal of H3K4me1 (top) and H3K27ac (bottom) occupancy related to human Notch1 on 7 human cell lines from ENCODE at UCSC Genome Browser.

B Single EPHB2^{high}OLFM4^{high} colon stem cells were transfected with either an empty vector control or CRISPR/Cas9 gRNAs. Shown are representative sequences from selected clones with indel mutations in the targeted region of the human NICD binding motif. Yellow box represents the putative binding sequence region, where red indicates indel mutations by CRISPR.

C EPHB2^{high}OLFM4^{high} colon stem cells were transfected with either an empty vector control or CRISPR/Cas9 gRNA and subsequently treated with DMSO, DAPT, or JAG1. Shown is ChIP-qPCR analysis of Notch1, indicating enrichment with NICD antibody compared with IgG control. The experiment was performed in triplicate and presented mean ± SEM (**$P \leq 0.01$; Student's *t*-test).

D Single EPHB2^{high}OLFM4^{high} colon stem cells were transfected with either an empty vector control or CRISPR/Cas9 gRNAs and cultured as organoids for 14 days. Top: Representative FACS plots for EPHB2 and OLFM4 expression and the percentage of EPHB2^{high}OLFM4^{high} stem cells for each condition. Bottom: FACS histograms indicating expression for the epithelial-specific cell marker EpCAM in the EPHB2^{high}OLFM4^{high} subset of cells for each condition.

 © 2017 The Authors

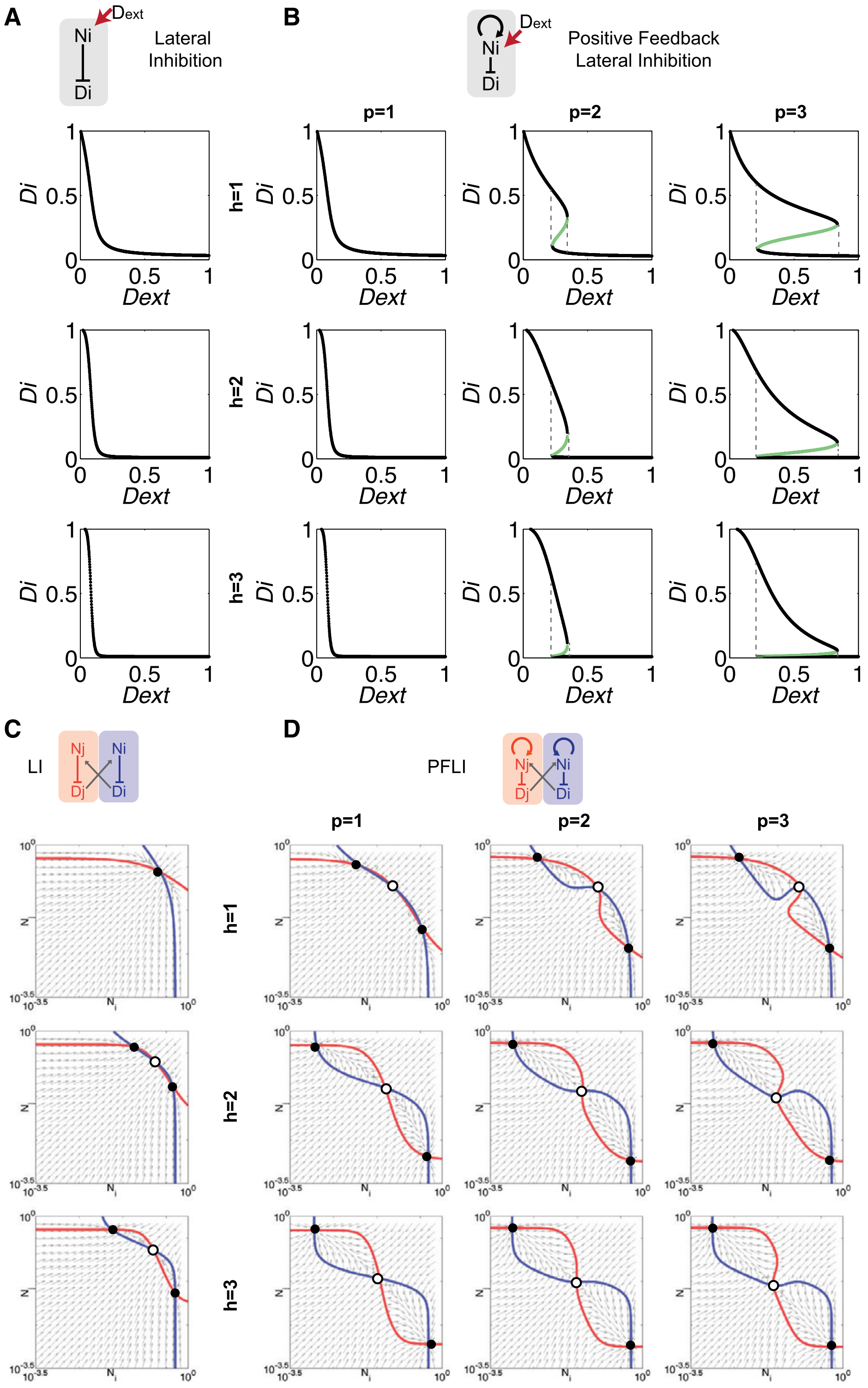

Figure EV5. Bistability of LI vs. PFLI.

A Dynamic analysis of the single-cell LI model. Internal Dll (D_i) vs. external Dll (D_{ext}) protein levels are plotted.

B Dynamic analysis of the single-cell PFLI model. Internal Dll (D_i) vs. external Dll (D_{ext}) protein levels are plotted. The black lines represent stable steady states, and the green lines represent unstable steady states.

C Phase portraits of the pair-cell LI model. h, Hill coefficient representing cooperativity of LI reaction. N_i, N_j and D_i, D_j refer to Notch and Dll levels in cells i and j, respectively.

D Phase portraits of the pair-cell PFLI model. h, Hill coefficient representing cooperativity of LI reaction. p, Hill coefficient representing cooperativity of the Notch positive feedback.

© 2017 The Authors

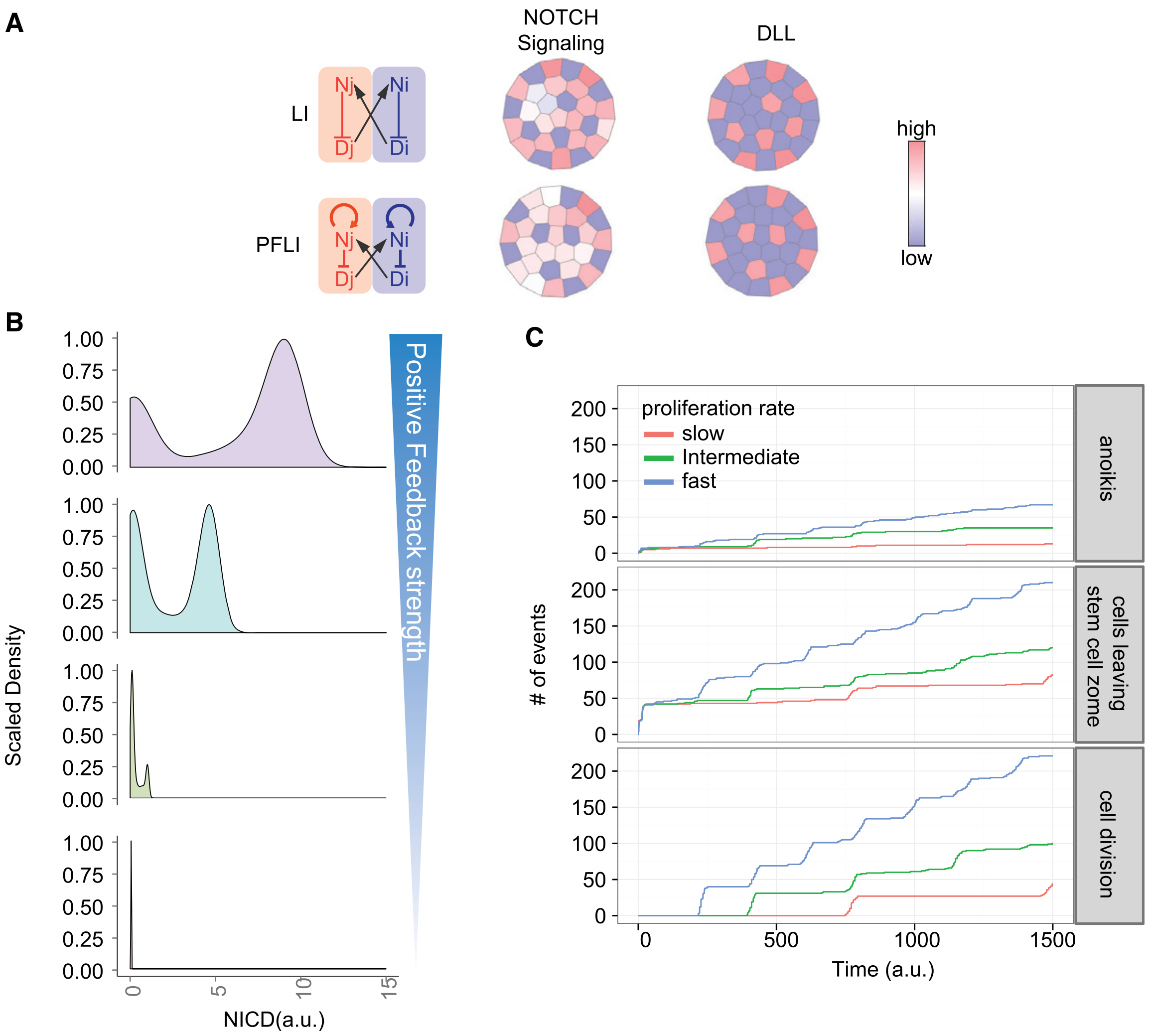

Figure EV6. Multicellular patterning simulation.

A Deterministic multicellular simulation of Notch expression patterns with LI and PFLI circuitry.

B Representative density plots of NICD distribution in the multiscale, stochastic model with gradual suppression of Notch positive feedback.

C Simulated stochastic cellular events (anoikis, cells leaving stem cell zone, and cell division) with increasing cell proliferation rates.

 © 2017 The Authors
