# Supplementary material for: A Notch positive feedback in the intestinal stem cell niche is essential for stem cell self‐renewal
Source: Mol Syst Biol. 2017 Apr 1;13(4):927. doi: 10.15252/msb.20167324 (PMC5408779; doi:10.15252/msb.20167324)
Supplement: Supplementary file 2 — Table EV1 [file MSB-13-927-s002.docx]

**Table EV1. Top 20 off target list of CRISPR/Cas9 gRNA design targeting mouse NICD binding on Notch1**

| Mouse | | | | | | Mouse | | | | | |
| --- | --- | --- | --- | --- | --- | --- | --- | --- | --- | --- | --- |
|  | **sequence** | **score** | **mismatches** | **UCSC gene** | **locus** |  | **sequence** | **score** | **mismatches** | **UCSC gene** | **locus** |
| mouse gRNA1 | TGCATGCATGGAAGGTGCCTGGG | 5.5 | 2MMs [2:19] | NM_001083937 | chrX:+7470984 | mouse gRNA2 | GTGCATGCATGGAAGGTGCCTGG | 7.2 | 2MMs [3:20] | NM_001083937 | chrX:+7470983 |
|  | TAAATGCAGGGAAGGTGCGAGAG | 0.9 | 3MMs [3:9:20] |  | chr6:-136722982 |  | GTACTTCCATGGAAGGTGCTTGG | 0.9 | 3MMs [5:7:20] |  | chr9:-14806709 |
|  | TATATGCAAGGAAGGTGCTTAAG | 0.6 | 3MMs [3:9:19] |  | chr5:-48985614 |  | CTACATCTAAGGAAGGTGCGTGG | 0.9 | 4MMs [1:7:8:10] |  | chr17:+71976788 |
|  | CAGCTGCATGGAAGGTGCCTGAG | 0.5 | 4MMs [1:3:4:19] |  | chr3:+53914229 |  | GTACATGCATGGCAGGTGTGAGG | 0.8 | 2MMs [13:19] |  | chr2:+34218417 |
|  | TACAGGATTGTAAGGTGCGTGGG | 0.5 | 4MMs [5:7:8:11] |  | chr5:+53072063 |  | GGTCATGCCAGGAAGGTGCGAGG | 0.8 | 4MMs [2:3:9:10] |  | chr4:+143116445 |
|  | TACATGTTTTAAAGGTGCGTTAG | 0.5 | 4MMs [7:8:10:11] |  | chr6:-138323516 |  | GGTCATGCCAGGAAGGTGCGAGG | 0.8 | 4MMs [2:3:9:10] |  | chr7:+73679070 |
|  | TACATGCGTGGCAGGTGCTTCAG | 0.4 | 3MMs [8:12:19] | NM_028326 | chr4:-62793576 |  | CTACATTCATGGAAGGTGTGCAG | 0.8 | 3MMs [1:7:19] |  | chr12:-39471772 |
|  | CACATGTCTGGAAGGTGCCTTGG | 0.4 | 4MMs [1:7:8:19] | NM_013529 | chr11:+49643346 |  | ATTTATGCATGCAAGGTGCGGGG | 0.7 | 4MMs [1:3:4:12] |  | chr11:+118560302 |
|  | TGAATGTATGGAAGGTGCCTGGG | 0.3 | 4MMs [2:3:7:19] |  | chr9:-22522074 |  | CTCCATCCCTGGAAGGTGCGTGG | 0.6 | 4MMs [1:3:7:9] |  | chr10:+70508744 |
|  | GACAGCCATGGAAGGTGCCTCAG | 0.3 | 4MMs [1:5:6:19] |  | chr5:+37699657 |  | ATAGATGTATGGAAGGTGGGCAG | 0.5 | 4MMs [1:4:8:19] |  | chr16:-11009464 |
|  | GATCTGCATGGAAGGTGTGTCAG | 0.3 | 4MMs [1:3:4:18] |  | chr12:-72478645 |  | ATGCTTGCATGGAAGGTGGGAAG | 0.5 | 4MMs [1:3:5:19] |  | chr14:+117883528 |
|  | TACCTGGATTGAAGGTGCTTGGG | 0.3 | 4MMs [4:7:10:19] |  | chr4:-148623595 |  | GAATATGGATGGAAGGTGTGTAG | 0.5 | 4MMs [2:4:8:19] |  | chr17:-63652471 |
|  | TGAGTGCATGGAAGGTGAGTGAG | 0.3 | 4MMs [2:3:4:18] | NM_007951 | chr12:-81741930 |  | GTGGATGGATGGAAGGTGTGAGG | 0.5 | 4MMs [3:4:8:19] |  | chr14:+35674903 |
|  | TAAAAGCCTGGAAGGTGAGTTAG | 0.3 | 4MMs [3:5:8:18] |  | chr5:-139833271 |  | ACACATACATGGAAGGTGCAAAG | 0.5 | 4MMs [1:2:7:20] |  | chrX:+49601612 |
|  | AACATGTGTGGAAGTTGCGTAGG | 0.3 | 4MMs [1:7:8:15] |  | chr2:+60056208 |  | GTGCTTCCATGGAAGGTGCTTGG | 0.5 | 4MMs [3:5:7:20] |  | chr9:-14806680 |
|  | TACATGCATGAGAGGTACGTAGG | 0.3 | 3MMs [11:12:17] | NM_172461 | chr9:-105225434 |  | GTAGACGCAGTGAAGGTGCGGGG | 0.4 | 4MMs [4:6:10:11] |  | chr11:-72862078 |
|  | TGCATGCATGGAAGGTCTGTTAG | 0.3 | 3MMs [2:17:18] |  | chr12:+11339840 |  | GTACCAGAATGGAAGGTGCATGG | 0.4 | 4MMs [5:6:8:20] |  | chrX:+11254343 |
|  | TACTTGCCTGGCAGGTGCCTGAG | 0.2 | 4MMs [4:8:12:19] |  | chr5:+11129376 |  | GCACATCCAGGGAAGGCGCGAAG | 0.4 | 4MMs [2:7:10:17] |  | chr5:+134659799 |
|  | TACTTGCCTGGCAGGTGCCTGAG | 0.2 | 4MMs [4:8:12:19] |  | chr5:+11772194 |  | GTACCTGGATTGAAGGTGCTTGG | 0.4 | 4MMs [5:8:11:20] |  | chr4:-148623596 |
|  | TACTTGCCTGGCAGGTGCCTGAG | 0.2 | 4MMs [4:8:12:19] |  | chr5:+10949486 |  | GTAGATGAATTGAAGGGGCGGAG | 0.3 | 4MMs [4:8:11:17] |  | chr15:-83732996 |
