# Supplementary material for: A Notch positive feedback in the intestinal stem cell niche is essential for stem cell self‐renewal
Source: Mol Syst Biol. 2017 Apr 1;13(4):927. doi: 10.15252/msb.20167324 (PMC5408779; doi:10.15252/msb.20167324)
Supplement: Supplementary file 3 — Table EV2 [file MSB-13-927-s003.docx]

**Table EV2. Top 20 off target list of CRISPR/Cas9 gRNA design targeting human NICD binding on Notch1**

| Human | | | | | | Human | | | | | |
| --- | --- | --- | --- | --- | --- | --- | --- | --- | --- | --- | --- |
|  | **sequence** | **score** | **mismatches** | **UCSC gene** | **locus** |  | **sequence** | **score** | **mismatches** | **UCSC gene** | **locus** |
| human gRNA1 | TTGTTTTGGGGGATCCCCGTTAG | 1.2 | 3MMs [2:3:17] |  | chr3:+37461420 | human gRNA2 | CAGTGCTTGAATTTCCCACGTGG | 1.7 | 3MMs [3:7:8] |  | chr3:-150800788 |
|  | TGTTTTTGGGGGATCCACTTAGG | 0.4 | 3MMs [3:17:19] |  | chr15:-50287896 |  | AGCTCCGGGTATTTCCCACGTGG | 1.3 | 4MMs [1:2:5:10] |  | chr7:+101650770 |
|  | CCCTTTTCGGGGATCCGGGTGGG | 0.3 | 4MMs [1:2:8:18] |  | chr16:+30572685 |  | CCATGCTGGCATTTCCCACGTGG | 0.9 | 4MMs [2:3:7:10] |  | chr15:+25059482 |
|  | TCCTTTTGGGGGATCCTTGTGAG | 0.3 | 3MMs [2:17:18] |  | chr17:-52574961 |  | CAGGGCAGGCATTTCCCACGTGG | 0.9 | 4MMs [3:4:7:10] |  | chr12:+128118403 |
|  | GGCATTTGGGGGCTCCTCGTCAG | 0.2 | 4MMs [1:4:13:17] | NM_001163438 | chrX:+134125391 |  | CACCGCCTGGATTTCCCACGGGG | 0.9 | 4MMs [4:7:8:10] |  | chr6:+143248339 |
|  | TGCTCTTGGTGGCTCCGCGCAGG | 0.2 | 4MMs [5:10:13:20] |  | chr1:-151812439 |  | CACGGGGTGTATTTCCCACGTAG | 0.8 | 4MMs [4:6:8:10] |  | chr20:-61762358 |
|  | TGGGTTTGGGGGCTCCACGTCAG | 0.2 | 4MMs [3:4:13:17] |  | chr10:-99735725 |  | CTCTGCGGAAATTTCCCAGGAGG | 0.7 | 3MMs [2:9:19] |  | chr13:+99738208 |
|  | TGCTCTTGGAGGTTCCCCGTAAG | 0.2 | 4MMs [5:10:13:17] |  | chr12:+2472362 |  | TATTGAAGGAATTTCCCACGTGG | 0.6 | 4MMs [1:3:6:7] |  | chr3:+37835032 |
|  | TGGTTTTCTGGGATCCGGGTCAG | 0.2 | 4MMs [3:8:9:18] |  | chr13:+112528885 |  | CAGTGAAAGAATTTCCCACGGGG | 0.5 | 4MMs [3:6:7:8] |  | chr7:+38149428 |
|  | GGCTTTTGGGGGATGCGTGTGAG | 0.2 | 3MMs [1:15:18] | NM_017896 | chr20:+61576826 |  | CACTCTAGGCATTTCCCACGAAG | 0.5 | 4MMs [5:6:7:10] |  | chr9:-121109553 |
|  | GGCTTCTGGAGGATCCGGGTAGG | 0.2 | 4MMs [1:6:10:18] |  | chr7:-4860807 |  | GACTTCGGGCATTTCCCAGGGAG | 0.5 | 4MMs [1:5:10:19] |  | chr19:-58990262 |
|  | GGCATTTGGGGGATCCCAGTGGG | 0.1 | 4MMs [1:4:17:18] |  | chr21:+44561395 |  | CACTGCTAGTGTTTCCCACGTGG | 0.5 | 4MMs [7:8:10:11] |  | chr2:+89573534 |
|  | TCCTCTTGGGGGATCCAAGTTGG | 0.1 | 4MMs [2:5:17:18] |  | chr17:-45933624 |  | CACTGCTAGTGTTTCCCACGTGG | 0.5 | 4MMs [7:8:10:11] |  | chr2:-89947551 |
|  | TGCATTTGAGGGATCCTCTTTGG | 0.1 | 4MMs [4:9:17:19] |  | chr1:-112292458 |  | CAATGTGGGCGTTTCCCACGTGG | 0.4 | 4MMs [3:6:10:11] |  | chr2:+8736393 |
|  | TGCTAACGGGGGAGCCGCGTCGG | 0.1 | 4MMs [5:6:7:14] |  | chr17:+16256191 |  | CAGTGCAGGCATTTCCCACTCAG | 0.4 | 4MMs [3:7:10:20] | NR_038365 | chr10:+133608391 |
|  | GGCTTTGGGGGGATCCTTGTCGG | 0.1 | 4MMs [1:7:17:18] |  | chr20:-36102802 |  | GACTGCGAGAGCTTCCCACGGGG | 0.4 | 4MMs [1:8:11:12] |  | chr1:+31201098 |
|  | TGCTTCGGGGGGATCCGCTGTGG | 0.1 | 4MMs [6:7:19:20] | NM_018197 | chr20:-50770007 |  | ATCTGCGGGACGTTCCCACGGAG | 0.4 | 4MMs [1:2:11:12] | NM_003970 | chr8:-2091265 |
|  | TGCTTTTGGGGGGTCCTTGTCAG | 0.1 | 3MMs [13:17:18] |  | chr8:-19543678 |  | CACAGCCAGAATCTCCCACGTGG | 0.4 | 4MMs [4:7:8:13] | NM_000064 | chr19:+6702155 |
|  | CGCTTTTGTGGGCTCTGCGTTAG | 0.1 | 4MMs [1:9:13:16] |  | chr15:-34804741 |  | GACTCCTGGAATTTCCCAGGAGG | 0.4 | 4MMs [1:5:7:19] |  | chr5:-10989133 |
|  | TCCTTTTGGGGGTTTCCCGTGGG | 0.1 | 4MMs [2:13:15:17] |  | chr5:+72999210 |  | TACTGCAGGCATTTCCCAAGGGG | 0.3 | 4MMs [1:7:10:19] |  | chr20:-21574231 |
