# Supplementary material for: A Notch positive feedback in the intestinal stem cell niche is essential for stem cell self‐renewal
Source: Mol Syst Biol. 2017 Apr 1;13(4):927. doi: 10.15252/msb.20167324 (PMC5408779; doi:10.15252/msb.20167324)
Supplement: Supplementary file 4 — Table EV3 [file MSB-13-927-s004.docx]

**Table EV3 : Parameter Table**

| Figure | Model | Parameters |
| --- | --- | --- |
| Fig 4A (LI) | Model 1 | β_n0_= 1, β_d0_= 0.01, β_n_= 0, β_d_= 1, β_N_= 1, β_D_= 1,α_n_= 1, α_d_= 1, α_N_= 1, α_D_= 1, k_d_= 0.1, h= 1 |
| Fig 4A (PFLI) | Model 1 | β_n0_= 0.04, β_d0_= 0.01, β_n_= 1, β_d_= 1, β_N_= 1, β_D_= 1,α_n_= 1, α_d_= 1, α_N_= 1, α_D_= 1 , k_p_= 0.01, k_d_= 0.1, h= 1, p= 3 |
| Fig 4B (LI) | Model 1 | β_n0_= 1, β_d0_= 0.01, β_n_= 0, β_d_= 1, β_N_= 1, β_D_= 1, α_n_= 1, α_d_= 1, α_N_= 1, α_D_= 1, k_d_= 0.1, h= [1-2] |
| Fig 4B (PFLI) | Model 1 | β_n0_= 0.1, β_d0_= 0.01, β_n_= 1, β_d_= 1, β_N_= 1, β_D_= 1,α_n_= 1, α_d_= 1, α_N_= 1, α_D_= 1 , k_p_= 0.0774, k_d_= 0.0167, h= [1-2], p= 1 |
| Fig 4C (LI) | Model 1 | β_n0_= 10^-2^-10^2^, β_d0_=10^-5^, β_n_=0, β_d_= 10^-2^-10^2^, β_N_= 1, β_D_= 1,α_n_= 1, α_d_= 1, α_N_= 1, α_D_= 1, k_d_= 0.1778, h= [1-3] |
| Fig 4C (PFLI) | Model 1 | β_n0_= 10^-5^, β_d0_=10^-5^, β_n_= 10^-2^-10^2^, β_d_= 10^-2^-10^2^, β_N_= 1, β_D_= 1,α_n_= 1, α_d_= 1, α_N_= 1, α_D_= 1, k_p_= 0.0774, k_d_= 0.0167, h= [1-3], p= 1 |
| Fig 4D | Model 1 | β_nm_=10, S_PF_=0-1, β_dm_ =100, S_LI_=1, β_N_= 1, β_D_= 1, α_n_= 1, α_d_= 1, α_N_= 1, α_D_= 1 , k_p_= various, k_d_= various, h= 3, p= 3 |
| Fig 4E (LI) | Model 3 | β_N_= 10, S_PF_=0, β_D_=100, S_LI_= 1,α_n_= 1, α_d_= 1, α_N_= 1, α_D_= 1, α_R_ = 1, k_d_= 0.1778, h= 3 |
| Fig 4E (PFLI) | Model 3 | β_N_= 10, S_PF_=1, β_D0_= 0, β_D_= 100,α_n_= 1, α_d_= 1, α_N_= 1, α_D_= 1, α_R_ = 1, k_n_=0.0042, k_d_= 0.015, h= 3, p=3 |
| Fig EV5A (LI) | Model 1 | β_n0_= 1, β_d0_= 0.0100, β_n_= 0, β_d_= 1, β_N_= 1, β_D_= 1,α_n_= 1, α_d_= 1, α_N_= 1, α_D_= 1,k_d_= 0.1,h= [1-3] |
| Fig EV5B (PFLI) | Model 1 | β_n0_= 0.04, β_d0_= 0.01, β_n_= 1, β_d_= 1, β_N_= 1, β_D_= 1,α_n_= 1, α_d_= 1, α_N_= 1, α_D_= 1 , k_p_= 0.01, k_d_= 0.1,h= [1-3],p= [1-3] |
| Fig EV5C (LI) | Model 1 | β_n0_= 1, β_d0_= 0.0100, β_n_= 0, β_d_= 1, β_N_= 1, β_D_= 1,α_n_= 1, α_d_= 1, α_N_= 1, α_D_= 1,k_d_= 0.1,h= [1-3] |
| Fig EV5D (PFLI) | Model 1 | β_n0_= 0.1, β_d0_= 0.01, β_n_= 1, β_d_= 1, β_N_= 1, β_D_= 1,α_n_= 1, α_d_= 1, α_N_= 1, α_D_= 1 , k_p_= 0.0774, k_d_= 0.0167, h= [1-3], p= [1-3] |
| Fig EV6A (LI) | Model 1 | β_n0_= 5, β_d0_= 10^-5^, β_n_= 0, β_d_= 5, β_N_= 1, β_D_= 1,α_n_= 1, α_d_= 1, α_N_= 1, α_D_= 1,k_d_= 0.1778,h= 3 |
| Fig EV6A (PFLI) | Model 3 | β_n0_= 10^-5^, β_d0_= 10^-5^, β_n_= 5, β_d_= 5, β_N_= 1, β_D_= 1,α_n_= 1, α_d_= 1, α_N_= 1, α_D_= 1 ,k_p_= 0.0115, k_d_= 0.015,h= 3,p= 1 |
| Fig EV6B | Model 3 | β_N0_= 0.1, β_N’_=[0.1,1,5,10], β_D0_= 0, β_D’_= 100,α_n_= 1, α_d_= 1, α_N_= 1, α_D_= 1, α_R_ = 1, k_n_=0.0042, k_d_= 0.015, h= 3, p=3, β_N0_= β_N_(1- S_PF_), β_N’_= β_N_(S_PF_), β_D0_= β_D_(1- S_LI_), β_D’_= β_D_(S_LI_) |
