# Supplementary figures and images for: A Notch positive feedback in the intestinal stem cell niche is essential for stem cell self‐renewal
Source: Mol Syst Biol. 2017 Apr 1;13(4):927. doi: 10.15252/msb.20167324 (PMC5408779; doi:10.15252/msb.20167324)

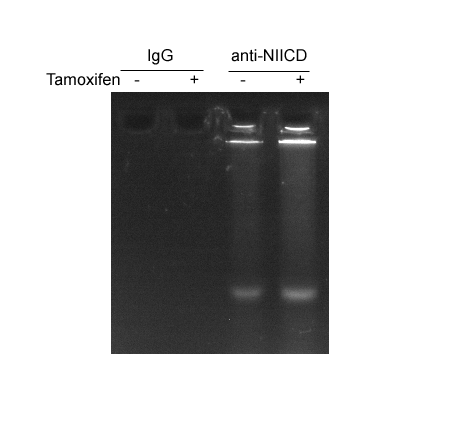

Supplement: Supplementary file 5 — Source Data for Expanded View [file MSB-13-927-s007.zip › SourceData_Figure_EV2/SourceData_Figure_EV2D/anti-NICD_and_IgG.tif]

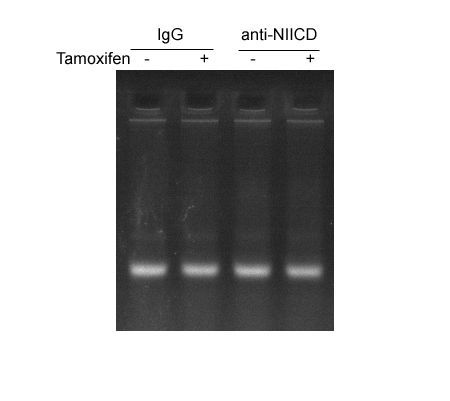

Supplement: Supplementary file 5 — Source Data for Expanded View [file MSB-13-927-s007.zip › SourceData_Figure_EV2/SourceData_Figure_EV2D/Input_control.tif]

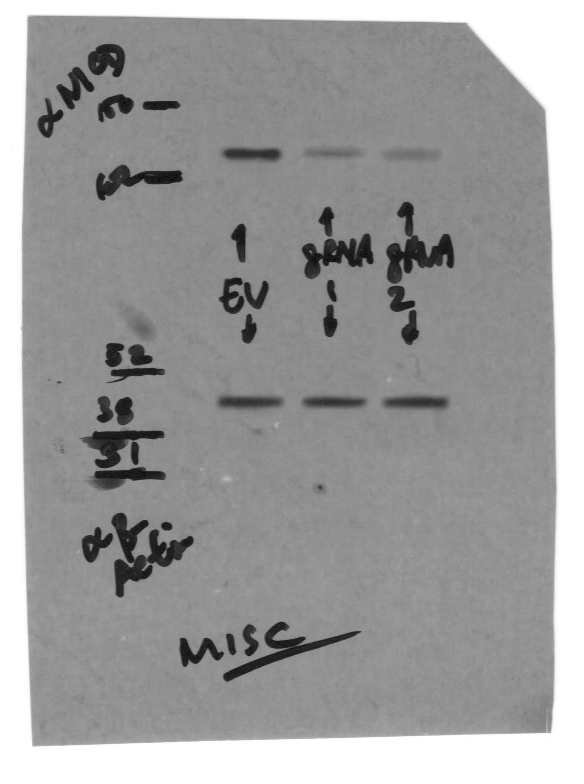

Supplement: Supplementary file 5 — Source Data for Expanded View [file MSB-13-927-s007.zip › SourceData_Figure_EV2/SourceData_Figure_EV2H/EV2H_-_Actin_.jpg]

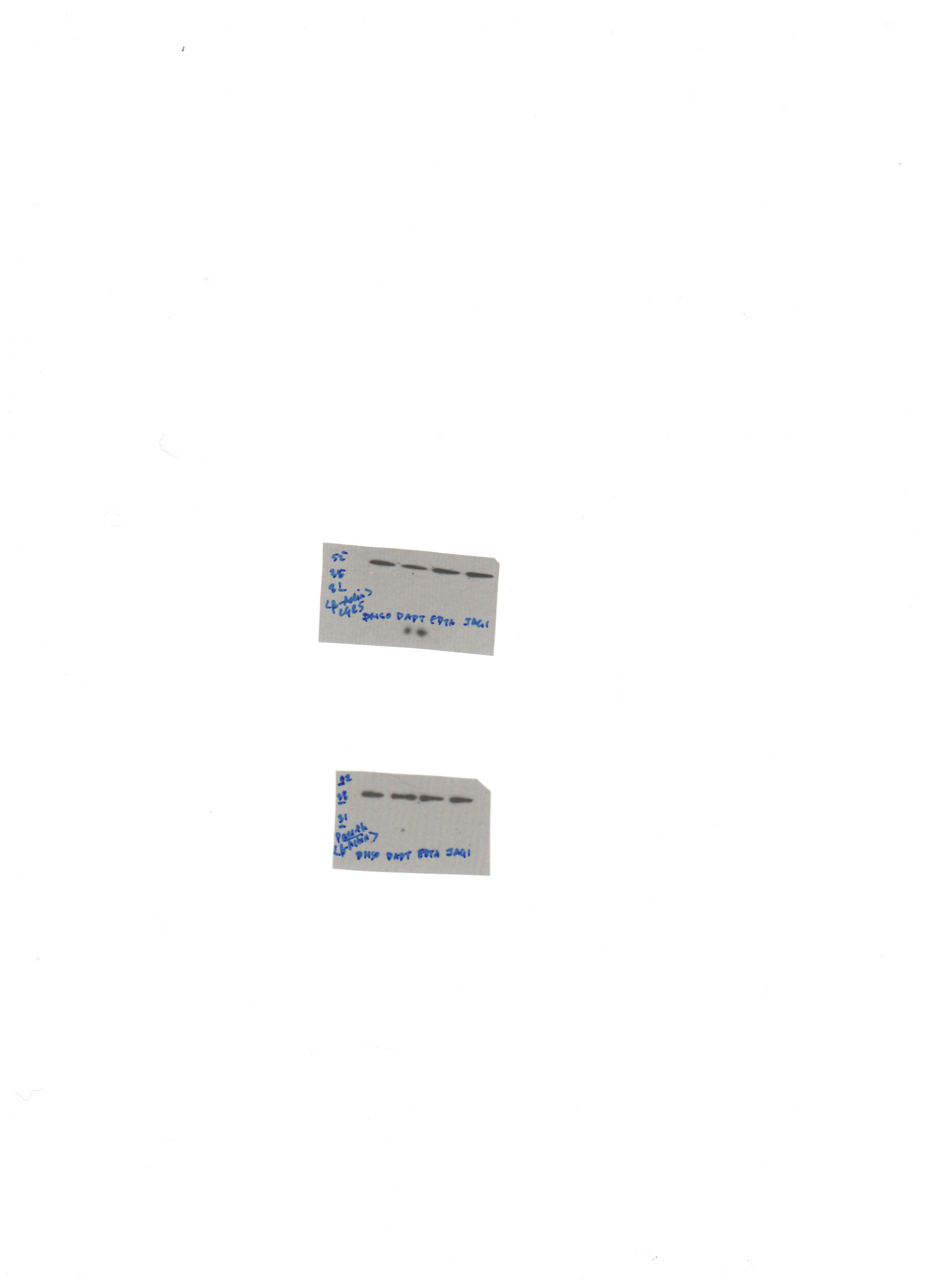

Supplement: Supplementary file 7 — Source Data for Figure 1 [file MSB-13-927-s005.zip › SourceData_Figure_1E/Fig_1_-_Actin.jpg]

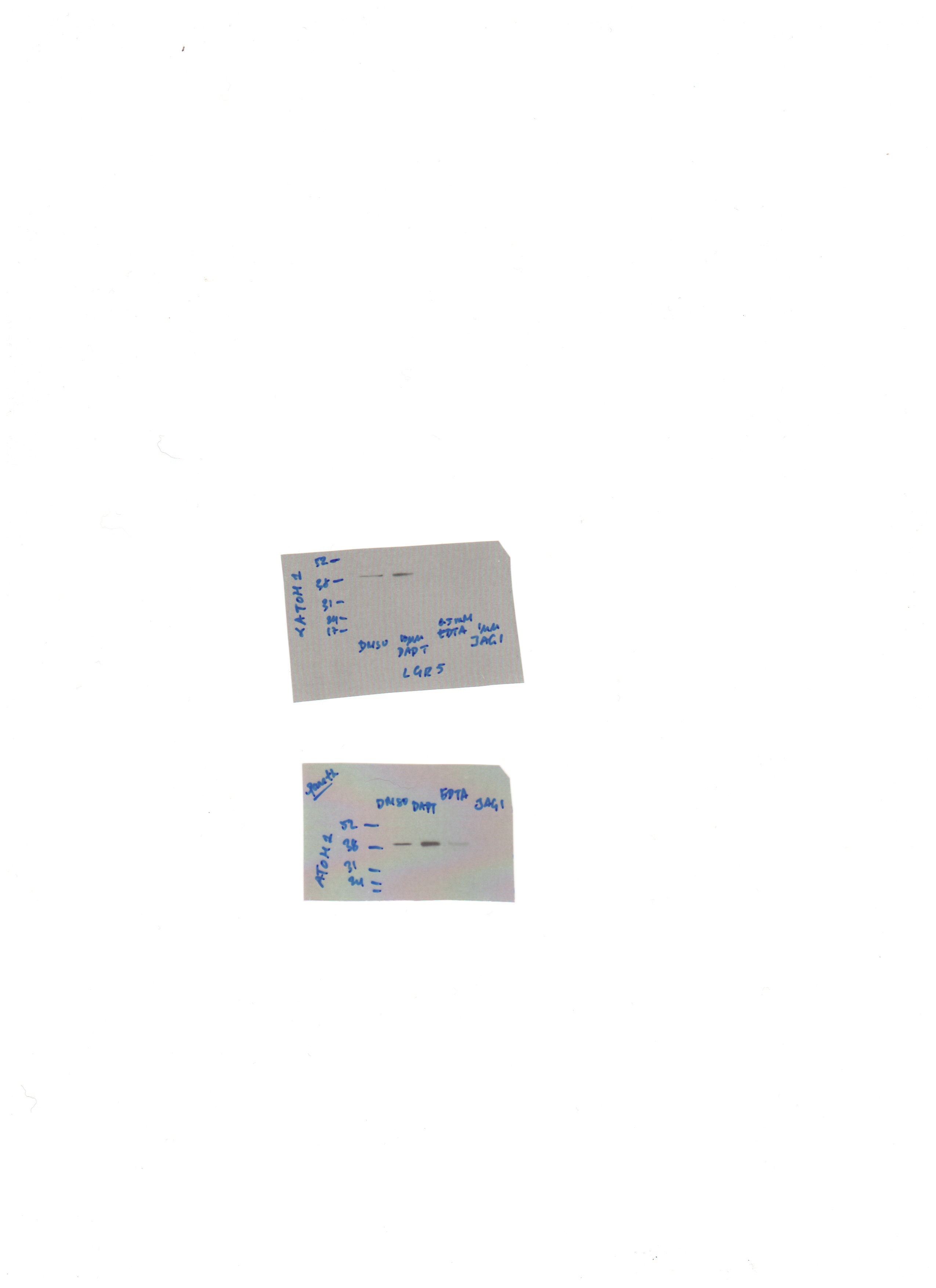

Supplement: Supplementary file 7 — Source Data for Figure 1 [file MSB-13-927-s005.zip › SourceData_Figure_1E/Fig_1_-_Atoh1.jpg]

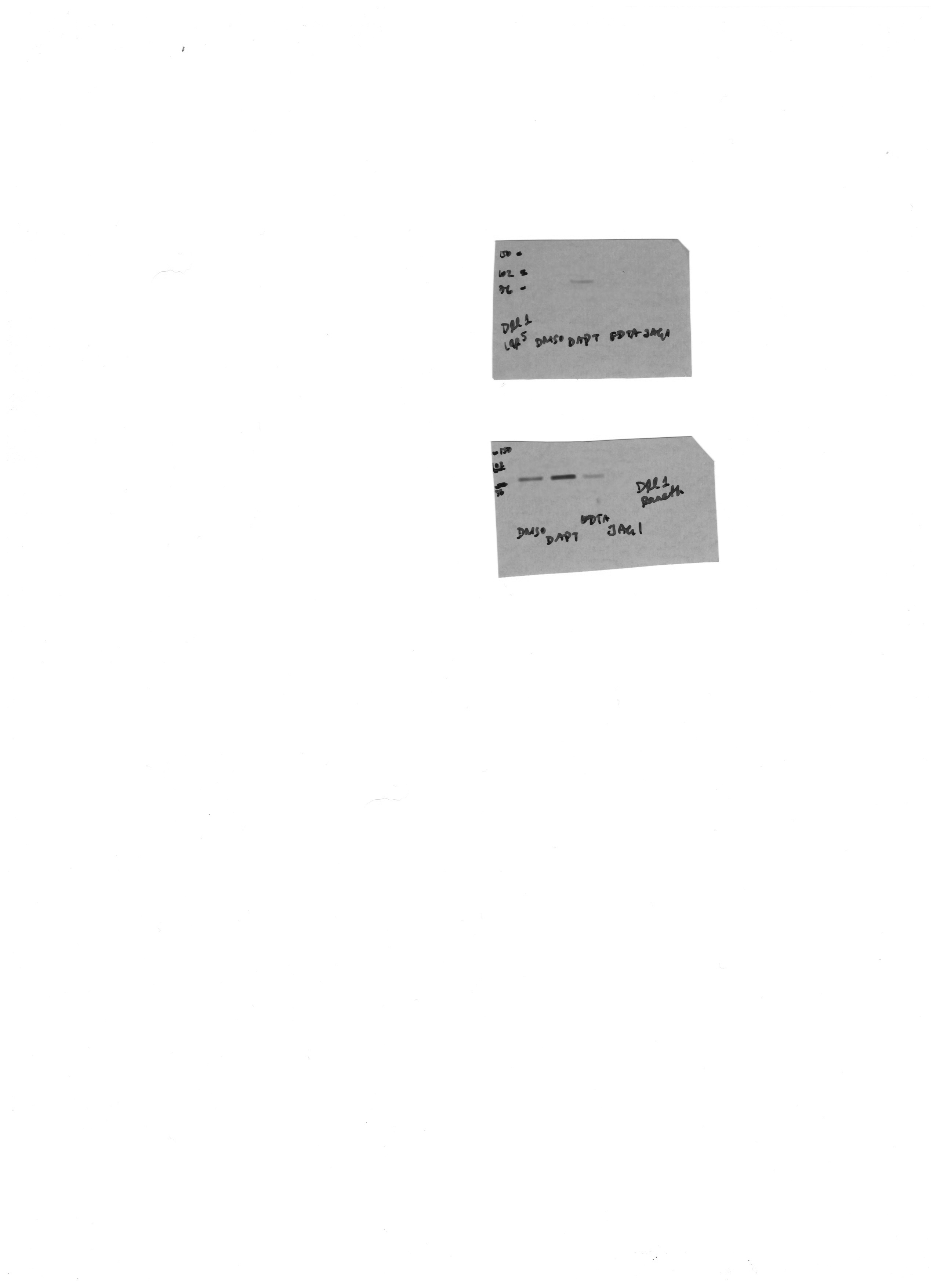

Supplement: Supplementary file 7 — Source Data for Figure 1 [file MSB-13-927-s005.zip › SourceData_Figure_1E/Fig_1_-_Dll1.jpg]

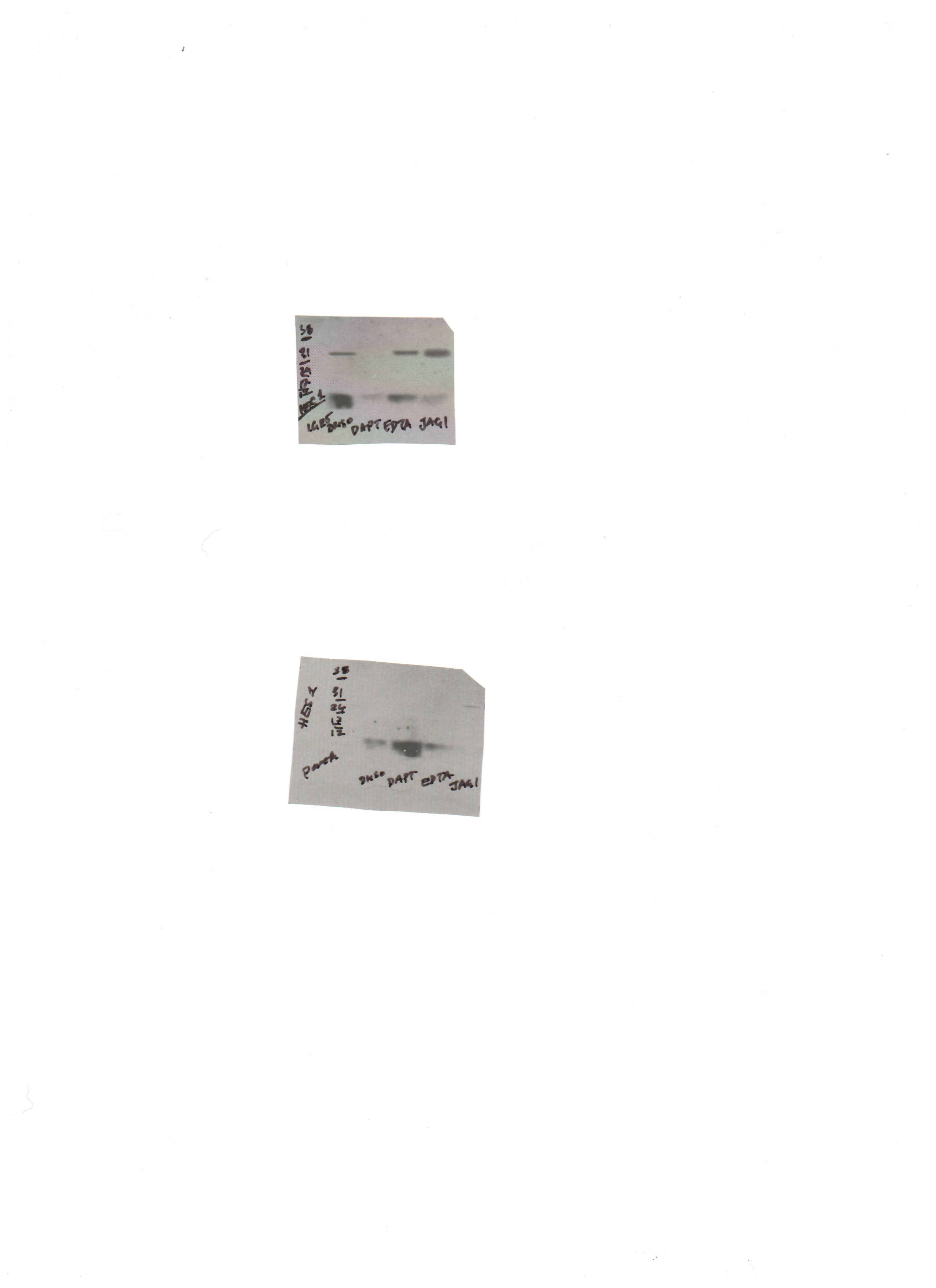

Supplement: Supplementary file 7 — Source Data for Figure 1 [file MSB-13-927-s005.zip › SourceData_Figure_1E/Fig_1_-_Hes1.jpg]

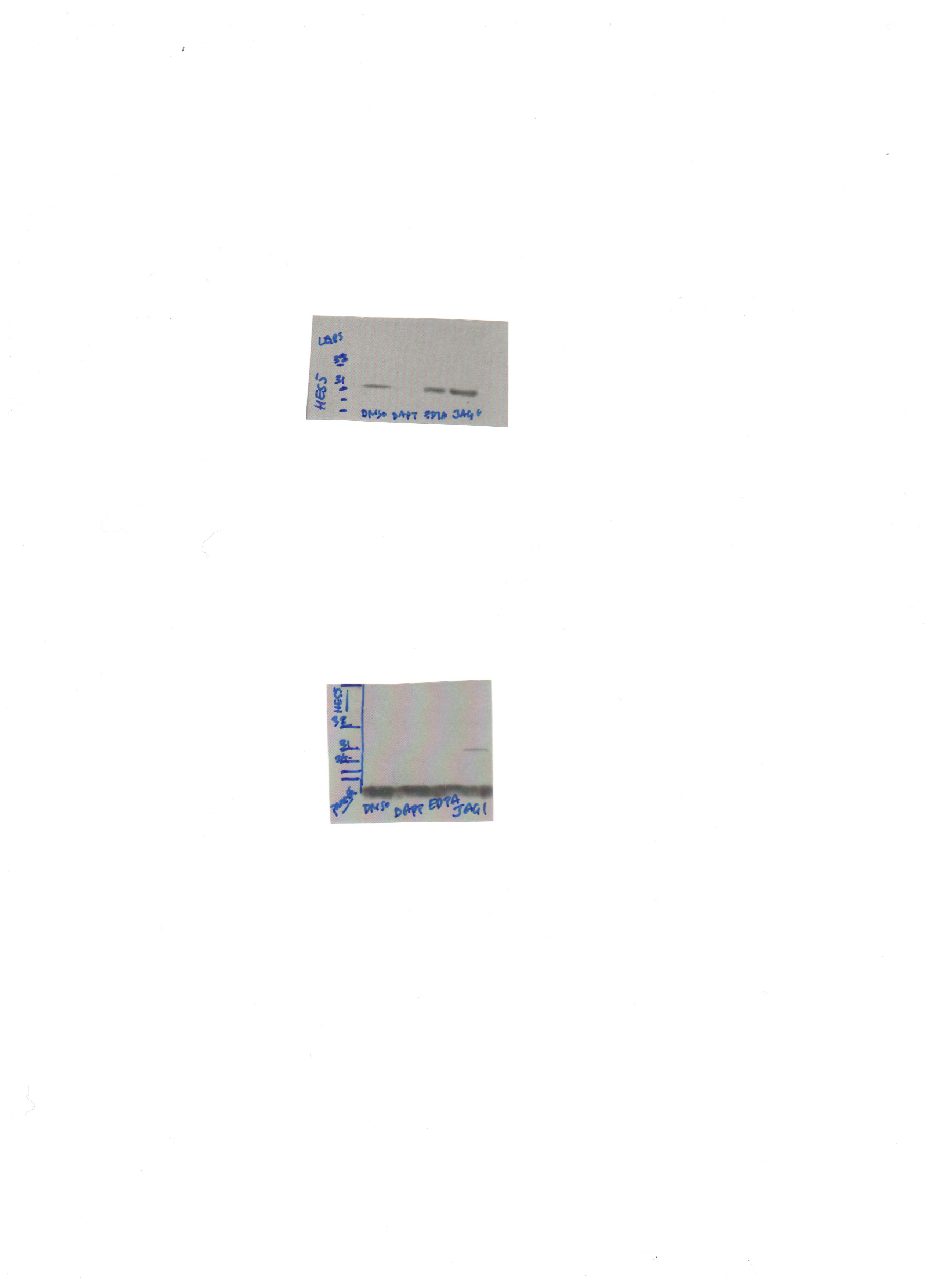

Supplement: Supplementary file 7 — Source Data for Figure 1 [file MSB-13-927-s005.zip › SourceData_Figure_1E/Fig_1_-_Hes5.jpg]

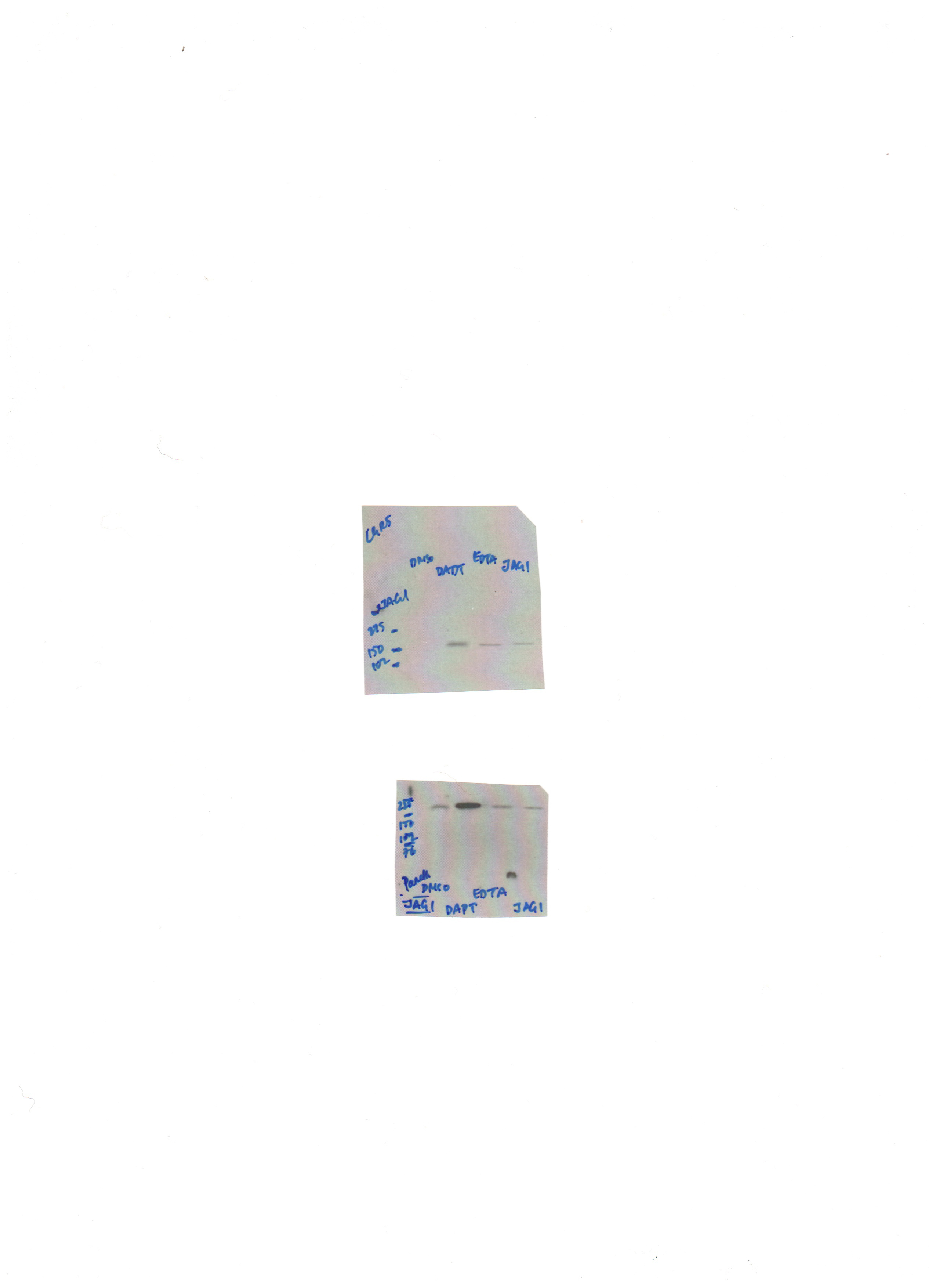

Supplement: Supplementary file 7 — Source Data for Figure 1 [file MSB-13-927-s005.zip › SourceData_Figure_1E/FIg_1_-_Jag1.jpg]

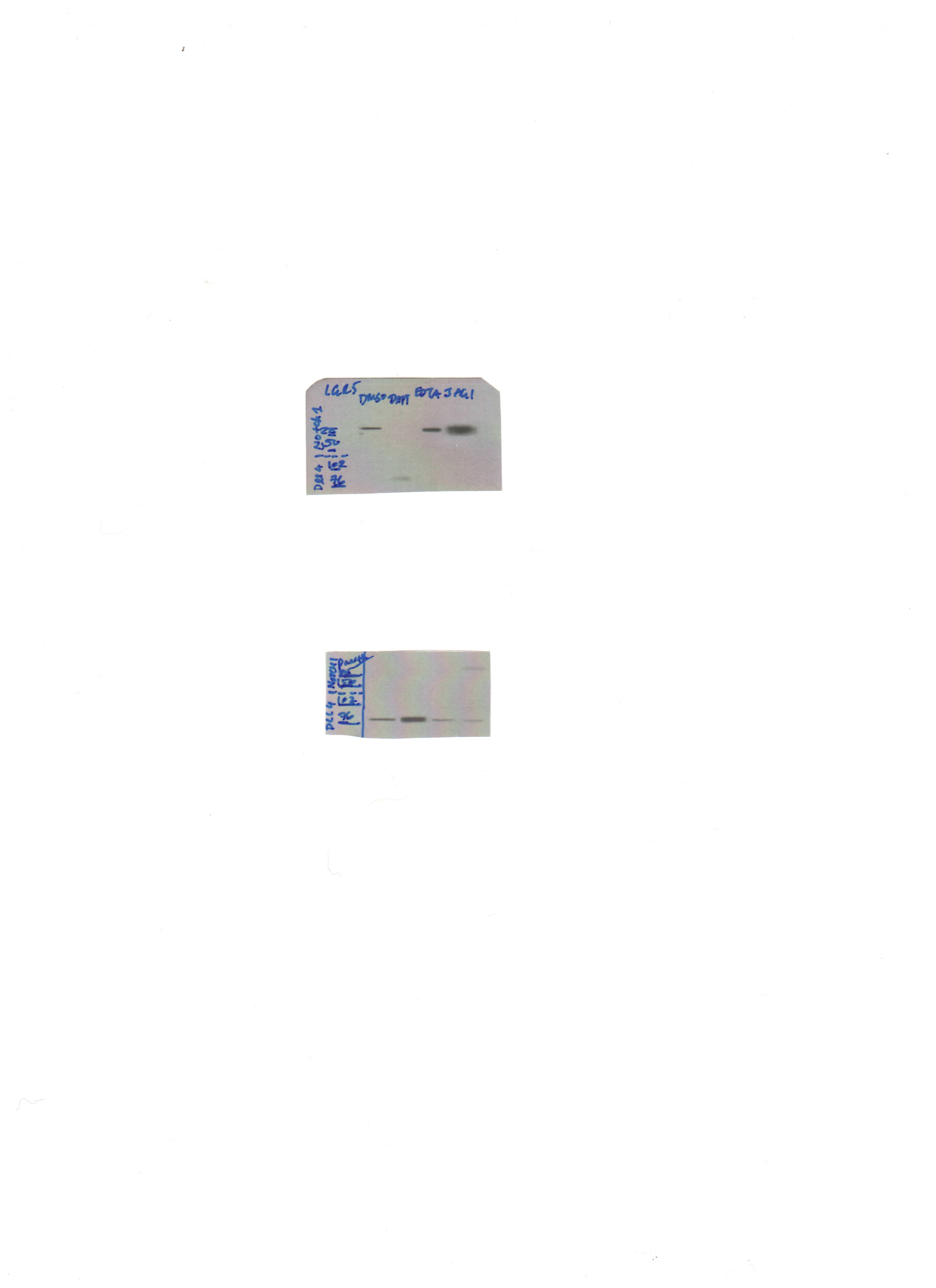

Supplement: Supplementary file 7 — Source Data for Figure 1 [file MSB-13-927-s005.zip › SourceData_Figure_1E/Fig_1_-_Notch1_Dll4.jpg]

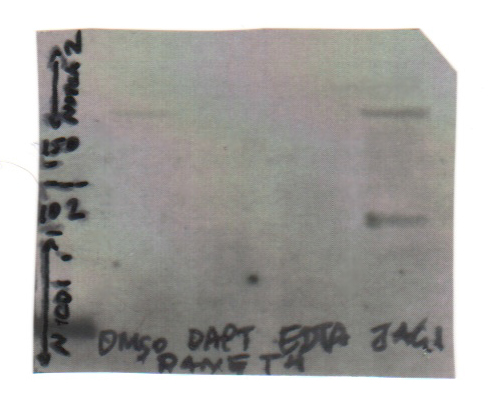

Supplement: Supplementary file 7 — Source Data for Figure 1 [file MSB-13-927-s005.zip › SourceData_Figure_1E/Fig_1_-_Notch2.jpg]

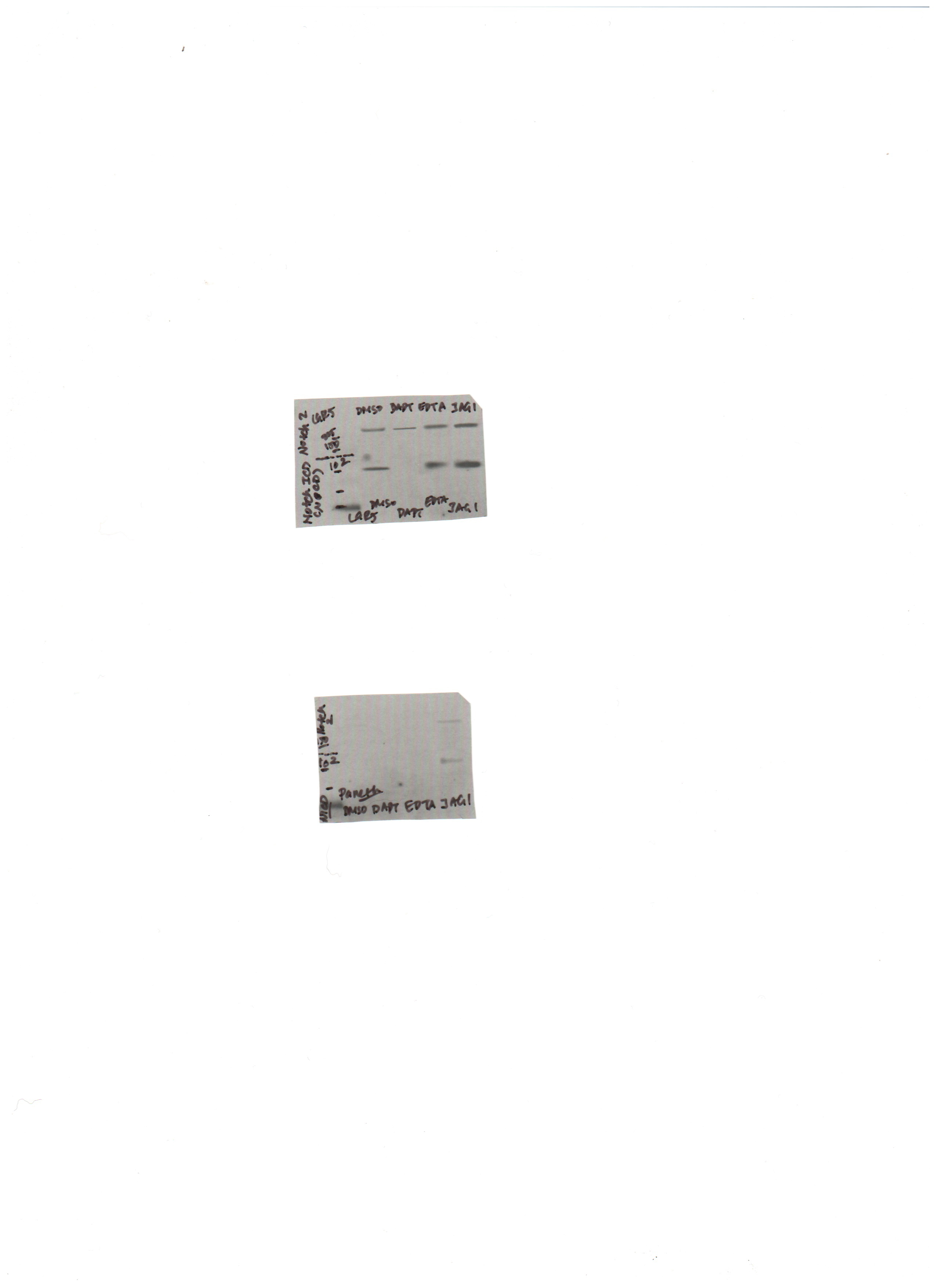

Supplement: Supplementary file 7 — Source Data for Figure 1 [file MSB-13-927-s005.zip › SourceData_Figure_1E/Fig1_-_Notch2_NICD.jpg]

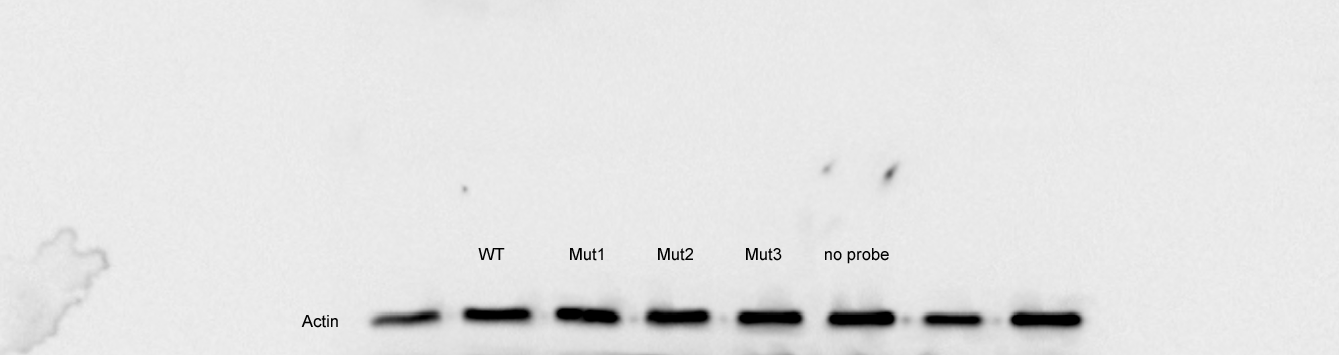

Supplement: Supplementary file 8 — Source Data for Figure 2 [file MSB-13-927-s006.zip › SourceData_Figure_2D/Actin.tif]

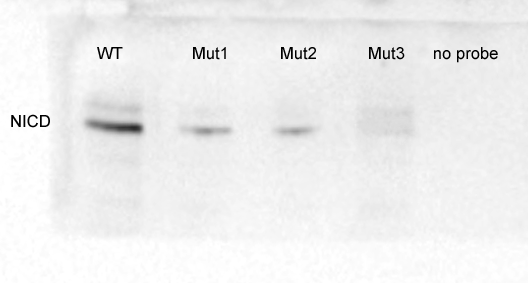

Supplement: Supplementary file 8 — Source Data for Figure 2 [file MSB-13-927-s006.zip › SourceData_Figure_2D/NICD.tif]
